# Molecular docking analysis of Withaferin A from Withania somnifera with the Glucose regulated protein 78 (GRP78) receptor and the SARS-CoV-2 main protease

**DOI:** 10.6026/97320630016411

**Published:** 2020-04-30

**Authors:** HV Sudeep, K Gouthamchandra, K Shyamprasad

**Affiliations:** 1 R and D Center for Excellence, Vidya Herbs Pvt. Ltd, #14A, Jigani I phase, Bangalore- 560 105, Karnataka, India

**Keywords:** COVID-19, GRP78, Mpro, Withaferin A

## Abstract

Design and development of an effective compound to combat COVID-19 is clearly critical in the current circumstances. Therefore, it is of interest to document the molecular docking analysis data of the cellular receptor Glucose regulated protein 78 (GRP78) with Withaferin A from Withania somnifera in the context of COVID-19 pandemic for further consideration. Here, we report the optimal interaction features of withaferin A, artemisinin, curcumin and andrographolide with the GRP78 receptor having low binding energies (-8.7, -7.89, -6.21 and -6.17 kcal/mol respectively) in this report. In order to gain additional insights, the interaction pattern of compounds with SARS-CoV-2 main protease (Mpro) was studied.

## Background

Novel coronavirus disease 2019 (COVID-19) caused by the severe acute respiratory syndrome coronavirus 2 (SARS-CoV-2) has been declared by the World Health Organization (WHO) as a global pandemic [1]. SARS-CoV-2 is an additional member of human coronaviruses (HCoVs) namely, NL63, 229E, OC43, HKU1, MERS and SARS. HCoVs have single stranded positive sense RNA that codes for Structural proteins and non-structural proteins. The structural proteins of HCoVs include spike protein, capsid, matrix and envelop. The non-structural proteins are proteases (nsp3, 5 and 12).

Development of therapeutic interventions to combat with COVID-19 pandemic depends largely on the understanding of coronavirus spike protein interaction with cell-surface receptors. SARS-CoV-2 invades the human cell via Angiotensin converting enzyme-2 (ACE-2) receptors [2,3].In addition to ACE-2 receptor other proteins present on the cell surface SIS known to be recognised by the virus for its entry. Glucose-regulated protein 78 (GRP78) primarily recognized as a protein present in endoplasmic reticulum with chaperone activity may translocate to the plasma membrane under cell stress [4,5]. Once localized to the cell surface GRP78 is prone to viral recognition through the substrate binding domain (SBD) subsequently facilitating the viral entry. Previously Chu et al. reported that GRP78 is a target cell surface receptor of the MERS-CoV spike protein [6]. More recently the binding pattern of spike protein of SARS-CoV-2 with GRP78-SBD has been predicted using computational approach [7].

Coronavirus main protease (Mpro/3CLpro) is another crucial molecular target in anti-CoV drug discovery research due to its indispensable requirement for viral replication and infection [8]. HCoV Mpro is a cysteine protease, which plays important role in the proteolytic activity during cleavage of viral polyprotein [9, 10]. Recently the crystal structure of coronavirus Mpro has been solved and publicly available in Protein data bank (PDB).

Several pharmacologically active principles from medicinal plants have been reported to exert antiviral activities [11,12, 13]. This study was conceived with a strategy of exploring the natural compounds which may impede the SARS-CoV-2 infection by blocking the viral entry into the host cell or inhibiting the viral polyprotein processing in the cell. In this direction, we have screened several small molecules of herbal origin and considered four major compounds withaferin A, artemisinin, curcumin and andrographolide fitting into our strategy. It is of interest to document the molecular docking analysis data of GRP78 with Withaferin A from Withania somnifera in the context of SARS-CoV-2 infection for further consideration.

## Materials and Methods

### Protein structures:

The crystal structures of GRP78 bound to ATP (PDB ID: 5E84) and COVID-19 3CLpro/Mpro (PDB ID: 6LU7) were downloaded in .pdb format from PDB database (http://www.rcsb.org/). Chain A of the respective proteins was used for macromolecule preparation.

The coordinates of PDB structures were prepared for molecular docking by removing the water ions and ligands using Python molecule viewer. The druggability pocket prediction was performed for the proteins using PockDrug-server [14,15].

### Ligand preparation:

We have used the potent pharmacologically active phytochemicals such as withaferin A, artemisinin, curcumin, and andrographolide in this study. The 3D structures of these compounds were obtained from Pubchem [16]. The drug-like properties of the molecules were determined using SWISSADME prediction [17]. The PRODRG server was used to minimise energy of natural compounds and 3D coordinates were prepared.

### Molecular docking:

The active site residues in the substrate-binding domain (SBD) of GRP78 were retrieved from the literature [18]. The active site amino acids of COVID-19 Mpro were determined using Discovery studio 4.5. AutoDock tool was utilized to generate grids, calculate dock score and evaluate the conformers of compounds bound in the SBD of GRP78, and active pocket of COVID-19 Mpro. The grid maps were centred with box size of 50 x 50 x 50 xyz points at active site residues of the proteins GRP78 (x = 34.386, y = 57.953, z = -29.261) and COVID-19 Mpro (x = -11.580, y = 16.427, z = 65.575), and generated with AutoGrid. As per genetic algorithm, all the torsions could rotate during docking. The Lamarckian genetic algorithm and the pseudo-Solis and Wets methods were applied for minimization, using default parameters [19].

## Results and discussion:

**Figure 1:**
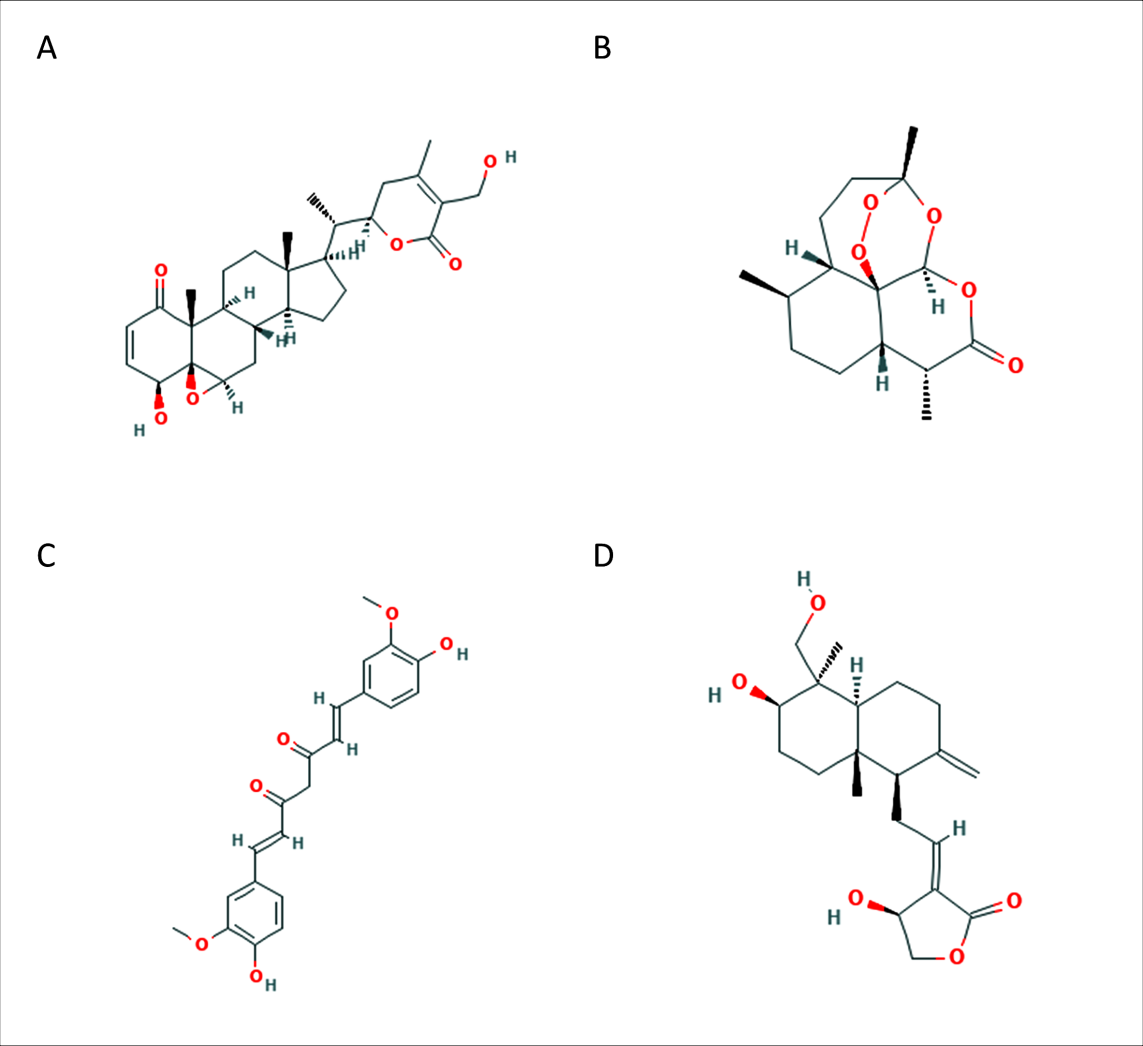
Chemical structure of Withaferin A (A), artemisinin (B), curcumin (C) and andrographolide (D).

**Figure 2:**
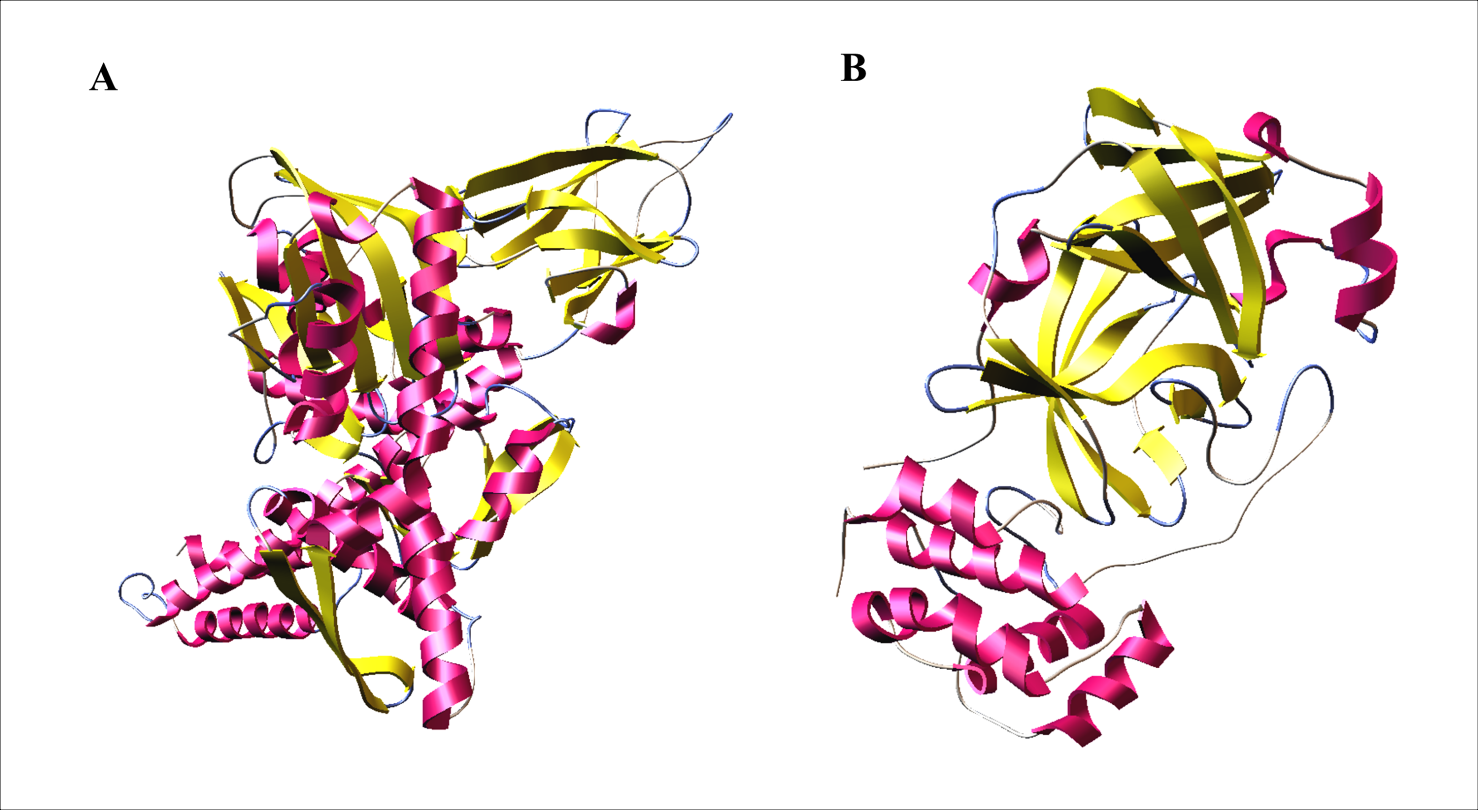
3D crystal structures of glucose regulated protein 78 (GRP78) (A) and COVID-19 Mpro (B).

In this study we have screened 25 natural compounds of plant origin for potential interaction with the possible therapeutic targets of SARS-CoV-2 infection. We could observe that few of them have shown appreciable binding affinities with GRP78 and COVID-19 Mpro. Hence, we have presented here the detailed molecular docking analysis of four promising candidates. Figure 1 shows the structures of compounds used for docking studies. We have initially considered predicting the drug like nature of the compounds based on Lipinski's rule of five (molecular weight, polar surface area, lipophilicity, hydrogen bonding and charge) as determined using SWISSADME. We found that all the tested molecules satisfied the rule of five in drug likeness (Table 1). Next, we have predicted the druggability of the protein pockets. The average druggability probability of GRP78 and COVID-19 Mpro pockets were 0.93±0.02 and 0.97±0.01 respectively indicating the likelihood of the pockets to be considered as druggable (Table 2).(Figure 2) shows the 3D structures of target proteins.

**Figure 3:**
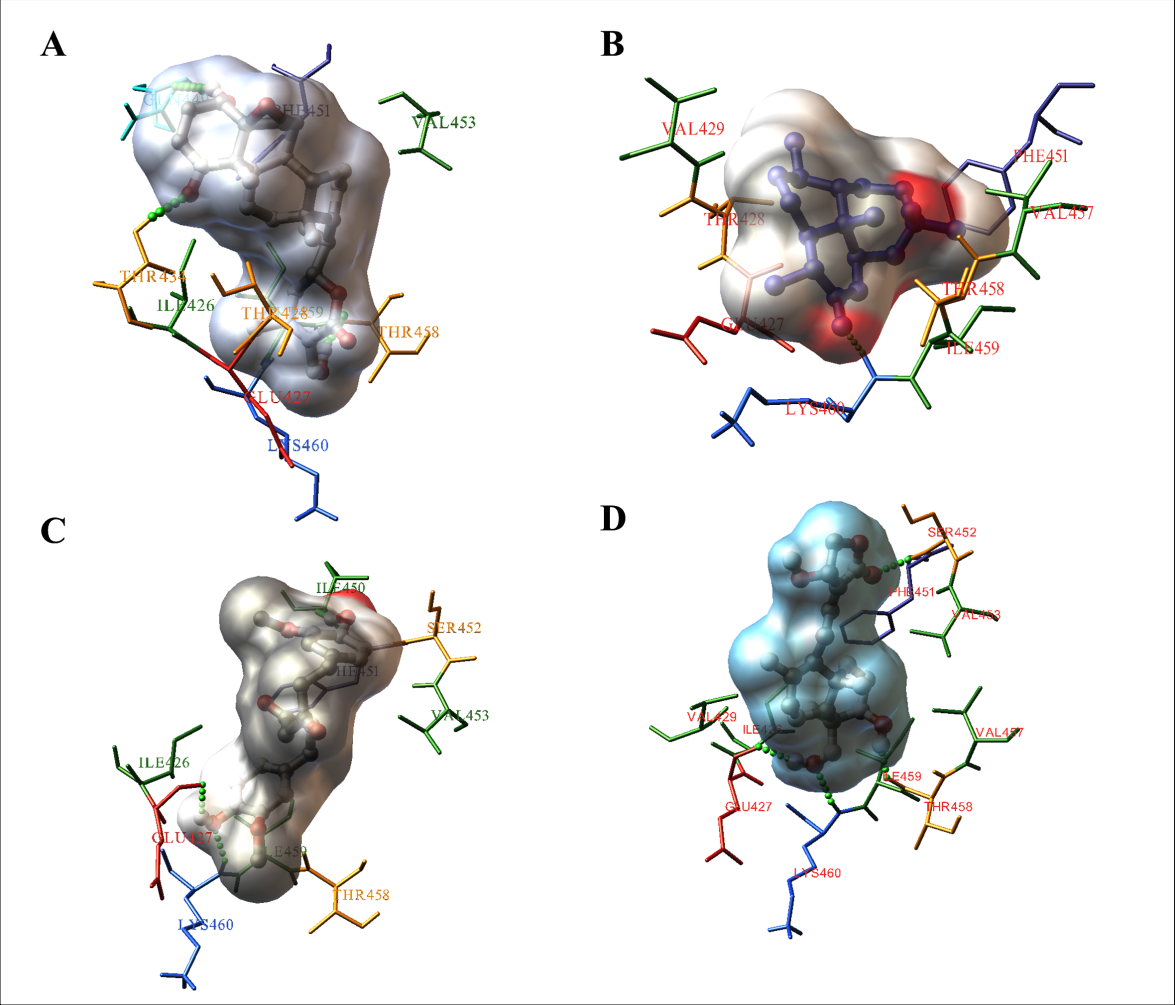
Interactions between the GRP78 with Withaferin A (A), artemisinin (B), curcumin (C) and andrographolide (D).

**Figure 4:**
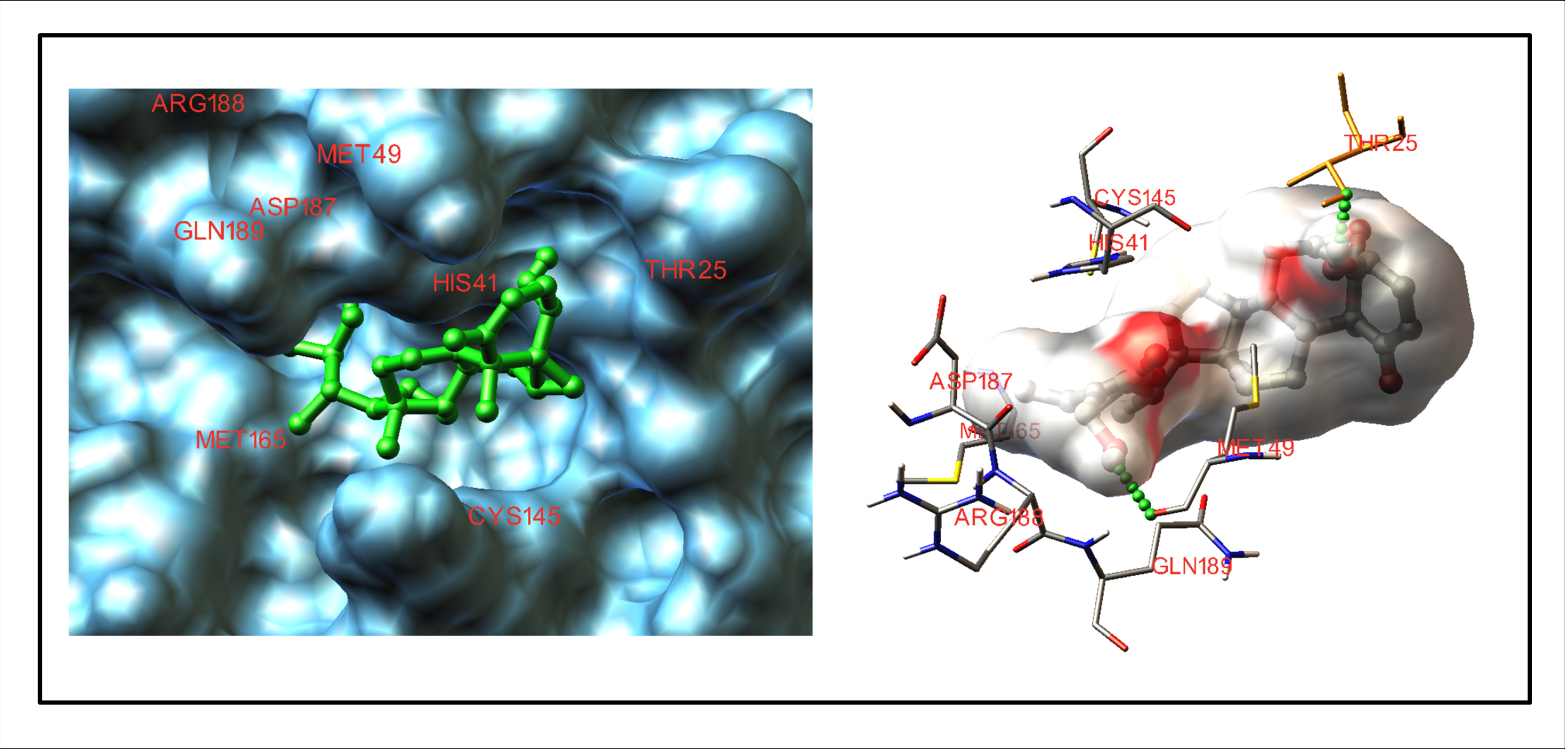
Interaction of withaferin A with the active site of COVID-19 Mpro (PDB: 6LU7).

We have determined the interactive pattern of test compounds with the SBD of GRP78 using molecular docking analysis. Interestingly, we found appreciable interactions between the active site residues of GRP78 and withanolide A with binding energy of -8.7 kcal/mol at its top-binding pose. Among the tested compounds withaferin A demonstrated strong interaction (Ki = 421 nM) with the binding site (Table 3). The molecule was found to have non-covalent interactions with Ile426, Thr428, Thr434 and Phe451 residues of GRP78 (Figure 3A). Further we have evaluated the binding prediction of the test compounds against the active site residues of COVID-19 Mpro (6LU7). The active site of 6LU7 consists of Thr24, Thr26, Asn142, Phe140, Gly143, His163, Glu166 and His172 residues. From our analysis, it was evident that withaferin A showed higher binding ability (Binding energy = -9.83 kcal/mol, Ki = 63 nM) into the active site of protein nevertheless; a noticeable interaction of all tested compounds was observed (Table 4). Figure 4 shows the top binding conformation of withaferin A with the COVID-19 Mpro active site.

**Figure 5:**
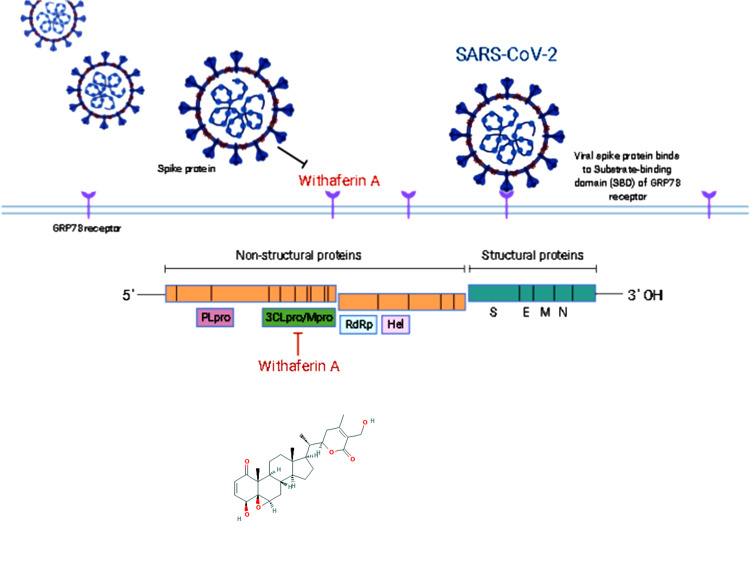
Schematic overview of proposed mechanism of action with which withaferin may inhibit COVID-19 infectivity. We hypothesise that withaferin A may exert its antiviral property against novel COVID-19 by either blocking the host cell receptor GRP78 or inhibiting the key viral protease 3CLpro/Mpro required for its replication in the host cell. PLpro: Pappain-like protease, 3CLpro/Mpro: 3C-like protease/main protease, RdRp: RNA dependent RNA polymerase, Hel: helicase; S: spike glycoprotein, E: envelop protein, M: membrane protein, N: Nucleocapsid protein.

In this study we have used the molecular docking tool to understand the interactions of natural products with plausible targets of SARS-CoV-2 infection such as host cell receptor GRP78 and COVID-19 Mpro. The ability of protein active sites to bind to drug-like molecules with extreme affinity is a major phase of target identification in drug discovery [20]. Our predictions indicated that the selected proteins showed high probability of druggability. Further it was found that the test compounds adhered to the Lipinski's rule of five indicating their improved permeability and drug likeness [21]. Docking studies revealed that withaferin A from Withania somnifera showed high affinity with GRP78-SBD. Recently, it has been reported that four regions of SARS-CoV-2 spike protein bind to the SBD of GRP78 for its entry into the host cell [7]. Here we speculate that withaferin A could bind to and block the receptor thereby inhibiting the viral entry. We have also performed docking analysis of the compounds as COVID-19 Mpro inhibitors. Proteases are often considered as key targets during the discovery of antiviral drugs as they play vital roles in viral replication and polyprotein processing [22] Interestingly, withaferin A was superior to the other tested compounds in its binding affinity with the protein active site.Withaferin A is a major active constituent of W. somnifera, medicinal herb greatly valued in Ayurveda for several medicinal properties including anti-viral activity [23,24]. This study hypothesises that withaferin A may exert its antiviral property against novel coronavirus SARS-CoV-2 by either blocking the host cell receptor or inhibiting the key viral protease required for its replication in the host cell. (Figure 5) illustrates the schematic overview of withaferin A with the molecular targets (Illustration was created using BioRender tool). Our predictions of its candidature in culminating SARS-CoV-2 infection may contribute to the use of natural products in antiviral therapy against the COVID-19 pandemic.

## Conclusions:

We have reported the optimal interaction features of withaferin A with the GRP78 receptor and SARS-CoV-2 Mpro in this study. Further research is required to validate the antiviral properties of withaferin A against COVID-19.

## Figures and Tables

**Table 1:** Drug likeliness of natural compounds

	Compounds			
Lipinski's rule of five	Withaferin A	Artemisinin	Curcumin	Andrographolide
Molecular weight (<500 Da)	470.6	282.33	368.38	350.45
MLog P (<4.15)	2.75	2.62	1.47	1.98
H-Bond donor (5)	2	0	2	3
H-Bond acceptor (<10)	6	5	6	5
Violation	0	0	0	0

**Table 2:** Pocket druggability prediction of the target proteins

Pockets	Vol. Hull*	Hydroph. Kyte*	Polar Res.*	Aromatic Res.*	Otyr atom	Nb. Res.*	Drugg Prob*	Standard Deviation
GRP78-SBD	6966.5	0.39	0.42	0.03	0	40	0.93	0.02
COVID-19 Mpro	28941.05	-0.11	0.54	0.13	0.01	161	0.97	0.01
Vol. Hull* = Volume Hull; Hydrophob. Kyte*= Hydrophobic Kyte; Polar Res.*= Polar Residues Proportion; Aromatic Res.*= Aromatic Residues Proportion (F,Y,H,W); Drugg Prob*= Druggability Probability; Nb. Res.*= Number of pocket residues

**Table 3:** Molecular docking analysis of compounds against GRP78 active site

Compound	Binding energy	Ligand efficiency	Inhibition constant (µM)	Intermolecular energy	VDW-H Bond Desolvation energy
Withaferin A	-8.7	-0.26	0.42	-9.89	-9.83
Artemisinin	-7.89	-0.39	1.66	-7.89	-7.81
Curcumin	-6.21	-0.23	28.17	-9.79	-9.74
Andrographolide	-6.17	-0.25	29.9	-7.96	-7.57

**Table 4:** Molecular docking analysis of compounds against 6LU7

Compound	Binding energy	Ligand efficiency	Inhibition constant (µM)	Intermolecular energy	vdW-H Bond Desolvation energy
Withaferin A	-9.83	-0.29	0.063	-11.02	-10.96
Artemisinin	-8.06	-0.4	1.24	-8.06	-7.95
Curcumin	-6.58	-0.24	15.15	-10.15	-9.99
Andrographolide	-6.49	-0.26	17.58	-8.28	-7.34
